# Abstract of Proceedings of Buffalo Medical Association

**Published:** 1866-02

**Authors:** Thos. M. Johnson

**Affiliations:** Secretary


### ART. II.—*Abstract of Proceedings of Buffalo Medical Association.*

TUESDAY EVENING, January 2d, 1866.

The Association met pursuant to adjournment, and was called to order by Dr. Congar, President pro tem.

Present—Drs. Congar, Miner, Shaw, Cronyn, Gay, Little, Boardman, Ring, Dayton, Johnson.

The minutes of the last meeting were read and accepted.

DR. MINER related the following case of operation for *impassable stricture*, which, he said, would illustrate several very important practical points in the management of similar cases, and was interesting mainly on that account: Herman Strootman, aged 14 years, came under his care July 6th, 1865, and gave the following history of his case: Fell, about three years since, through an iron grating, striking heavily upon the perineum, which caused great swelling, pain and difficult urination. The swelling abated gradu ally, and no instrument was passed into the bladder at that time. In about six months there came gradual difficulty in passing urine, which increased until it became almost an impossibility. Instruments could not be passed when the attempt was made, and the warm bath offered all the relief he could obtain. A great many unsuccessful attempts were made to relieve the stricture, until finally he was admitted to the Hospital of the Sisters of Charity, and was there treated several weeks during the previous winter, but without relief; daily attempts to pass instruments into the bladder having failed, he returned home, where abscesses formed in the perineum and opened spontaneously. Urine also escaped through the openings thus made for a few weeks, and then the passage closed, urine passing constantly by drops the natural way, with great effort and constant pain, often so great as to compel resort to the warm bath, which always afforded some relief. Was treated, after leaving the hospital, by a great many physicians, with medicines only. At the time of application to Dr. M., the urine could be passed only by drops and a resort to the warm bath three and four times daily. The clothes and bed-clothes were wet by the constant discharge, which was accompanied by strong involuntary effort; the bladder having taken on very much the action of a contracting uterus. The cries and screams of the patient were terrific, and life had become almost unendurable. Attempts were made to pass the smallest sized bougies, repeating the efforts for three or four days, leaving the instrument in the stricture as long as possible, with the hope of forcing some dilatation. A very small flexible English catheter would engage quite frequently in the contracted portion, at length in attempting to withdraw it, it separated, leaving about half an inch of the instrument inserted in the stricture. Urination was now impossible, and an operation unavoidable. Assisted by his pupils, the operation for impassable stricture was successfully made in the usual manner, with less difficulty than is common in such cases. Dr. Mixer arrived in time to afford assistance in the dressings, and observe the thickened condition of the strictured portion. Fully an inch in length of the membranous portion of the urethra was a dense and firm, fibrous structure, of at least half an inch in thickness before reaching the centre. The catheter which had been broken was first removed, and beyond its point it was impossible to trace the track of the urethra. Fortunately, the healthy portion posterior to the stricture was considerably dilated, as is common in such cases, and was readily found after dividing the diseased portions. A lead catheter was introduced and retained until the wound healed over it, which required but a few days. It was then removed, and carefully replaced during the first few days, and the case dismissed with the direetion that the catheter be removed and cleaned, and only worn a part of the time—nights only. Six weeks after this he met the patient upon the street, and upon careful inquiry found that the instrument had never been removed, the boy thinking himself all right, and better off to remain without further trouble. After explaining the necessity of so doing, the patient was brought for change of instruments, or removal of the one which had done such admirable service. Attempts were made to remove the instrument, but they were unsuccessful. The nature of the case being suspected, chloroform was given to complete anæsthesia, and after considerable gradual and increasing force had been applied, the instrument was removed. Upon the end which had rested in the bladder was closely adherent a round dense secretion—a stone, an inch in length and something more than half an inch in diameter, which had retained its connection with the instrument, and sustained the force which had dilated the urethra and allowed the passage of a substance at least twice the size of the natural channel. Since this time the boy has passed once or twice a week a full sized catheter, and reports himself perfectly well.

The practical points are too obvious to require mention. Under all ordinary circumstances, to have an instrument break in a stricture would be exceedingly embarrassing, and suggests care in using the flexible frail instruments for such purposes. In this case, since the operation for perineal section had nearly been decided upon previous to the accident, it was in no way important, and only hastened a measure which otherwise would have been very soon adopted. The natural hesitancy which every surgeon feels in attempting this operation who has ever made it, had operated to cause delay and attempts to avoid it; and perhaps the necessity was not made certain any too early. Comparatively few surgeons have attempted without guide to trace the obliterated track of the urethra and find it again near the bladder. It is justly considered the most difficult and uncertain operation in surgery, and, withal, attended with great danger. Whoever has had experience, would not unhesitatingly attempt it, and although he had twice before successfully made it, he hoped occasion wonld never again demand of him such an operation.

The danger of leaving a catheter in the bladder for a long time without removal, was forcibly illustrated in this case, as also was the dilatibility of the urethral canal in its normal condition. If a stone of that size can be slowly and gradually extracted through the urethra without serious injury to its mucous membrane or walls, it seems probable that practical deductions might be gathered from such facts, that possibly stones which have heretofore been crushed in the bladder might have been removed entire through the urethra. The results of this case were remarkably satisfactory. The boy, whose life had been one of untold misery, was restored to health, vigor and comfort.

Dr. Cronyn said he was much interested in the case reported by Dr. Miner, and thought the doctor entitled to credit for his success in the operation. Had often seen the deposit spoken of, and thought it usual.

Dr. C. L. Dayton presented two specimens of tape worm, and believed that the head had in both cases been expelled with the body, but had not made a microscopical examination. The first patient was a man of middle age, and the second a young lady of eighteen. Gave ℥i of kosso in ʒi doses every four hours in each case.

Dr. CRONYN remarked that the cases related by Dr. Dayton brought to mind the superior value of kosso. Had lately given it to a patient who had taken other remedies in vain. The patient soon passed thirty feet of tape worm, and a few days after resort was again had to the remedy, with the result of the passage of about one hundred feet.

Dr. DAYTON finds that kosso often causes vomiting unless preceded by a cathartic, the operation of which will almost invariably prevent vomiting.

Dr. MINER thinks that kosso is usually given in too large doses, and believes that half the usual quantity is sufficient.

Dr. GAY wished to call the attention of the association to the use of sub-sulphate of iron in diphtheritic diseases. He uses this preparation in preference to the other ferruginous preparations in this disease, and thinks it will be found superior to them. His formula for the preparation and use is—sol. subsulphate ferri gtt xx—xxx syr. simplex. aqua mentha aa ℥i. Dose, a teaspoonful every two hours for children. Dr. Gay called the attention of the association to this subject at the last December meeting, but was then incorrectly reported as making the above remarks in regard to the muriated tinct. ferri instead of the sol. sulphate.

Reports on prevailing deseases being called for, variola, varioloid, diphtheria, typhoid and intermittent fevers were reported as prevailing.

Dr. BOARDMAN reported that he had found revaccination to take more readily than usual, that persons on whom revaccination took well from two to five years ago, take revaccination readily now.

Dr. JOHNSON had remarked the same fact, and had seen several marked cases of the kind alluded to.

Dr. CRONYN would like to inquire if sarrasenia had been prescribed in variola by members present.

Dr. MINER said he had not used it, and believed it entirely useless in that class of diseases, and thought no one, after having read the reports of it in the journals, would think it worthy a trial.

On motion, the Association returned to voluntary communications, when a discnssion was had upon the merits of sarracenia in variola.

Dr. Cronyn spoke at considerable length and very favorably of it in that class of diseases, and will at no distant day report a number of cases in point.

Dr. Dayton tried it thoroughly when health physician and discarded it, believing it useless, and supposed that to be the general belief.

Dr. Ring, Chairman of the Auditing Committee, reported that the Committee found Dr. J. A. Peters' accounts correct. Report accepted.

Dr. Gay moved that the thanks of the Association be tendered to Dr. Peters for the efficient and acceptable manner in which he has performed the duties of Secretary. Carried.

Adjourned. Thos. M. Johnson, *Secretary.*

